# Investigation of Dextran-Coated Superparamagnetic Nanoparticles for Targeted Vinblastine Controlled Release, Delivery, Apoptosis Induction, and Gene Expression in Pancreatic Cancer Cells

**DOI:** 10.3390/molecules25204721

**Published:** 2020-10-15

**Authors:** Salim Albukhaty, Sharafaldin Al-Musawi, Salih Abdul Mahdi, Ghassan M. Sulaiman, Mona S. Alwahibi, Yaser Hassan Dewir, Dina A. Soliman, Humaira Rizwana

**Affiliations:** 1 Department of Basic Sciences, College of Nursing, University of Misan, Maysan 62001, Iraq; albukhaty.salim@uomisan.edu.iq; 2 Faculty of Biotechnology, Al-Qasim Green University, Babylon 51013, Iraq; drsalih@biotech.uoqasim.edu.iq; 3 Department of Applied Sciences, University of Technology, Baghdad 10066, Iraq; 100135@uotechnology.edu.iq; 4 Department of Botany and Microbiology, College of Science, King Saud University, P.O. Box 2455, Riyadh 11451, Saudi Arabia; malwhibi@ksu.edu.sa (M.S.A.); dsoliman@ksu.edu.sa (D.A.S.); hrizwana@ksu.edu.sa (H.R.); 5 College of Food and Agriculture Sciences, King Saud University, Riyadh 11451, Saudi Arabia; ydewir@ksu.edu.sa; 6 Faculty of Agriculture, Kafrelsheikh University, Kafr El-Sheikh 33516, Egypt

**Keywords:** superparamagnetic iron oxide nanoparticles, vinblastine, dextran, folic acid, apoptotic cells, gene expression, pancreatic cancer, nanostructure

## Abstract

In the current study, the surface of superparamagnetic iron oxide (SPION) was coated with dextran (DEX), and conjugated with folic acid (FA), to enhance the targeted delivery and uptake of vinblastine (VBL) in PANC-1 pancreatic cancer cells. Numerous analyses were performed to validate the prepared FA-DEX-VBL-SPION, such as field emission scanning transmission electron microscopy, high-resolution transmission electron microscopy, dynamic light scattering (DLS), Zeta Potential, Fourier transform infrared spectroscopy, and vibrating sample magnetometry (VSM). The delivery system capacity was evaluated by loading and release experiments. Moreover, in vitro biological studies, including a cytotoxicity study, cellular uptake assessment, apoptosis analysis, and real-time PCR, were carried out. The results revealed that the obtained nanocarrier was spherical with a suitable dispersion and without visible aggregation. Its average size, polydispersity, and zeta were 74 ± 13 nm, 0.080, and −45 mV, respectively. This dual functional nanocarrier also exhibited low cytotoxicity and a high apoptosis induction potential for successful VBL co-delivery. Real-time quantitative PCR analysis demonstrated the activation of *caspase-3*, *NF-1*, *PDL-1*, and *H-ras* inhibition, in PANC-1 cells treated with the FA-VBL-DEX-SPION nanostructure. Close inspection of the obtained data proved that the FA-VBL-DEX-SPION nanostructure possesses a noteworthy chemo-preventive effect on pancreatic cancer cells through the inhibition of cell proliferation and induction of apoptosis.

## 1. Introduction

**Figure 1:**
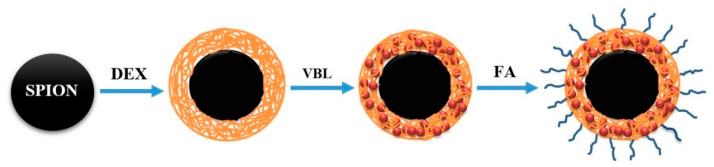
Schematic illustration of the procedure used for the synthesis and formulation of the FA-DEX-VBL-SPION nanostructure.

Vinblastine (VBL) is a natural alkaloid extracted from the *Vinca rosea Linn* plant. VBL binds to tubulin and inhibits the formation of microtubules, causing disruption of the assembly of mitotic spindles and the arrest of tumor cells in the M phase of the cell cycle [1]. VBL, which is marketed under the Velban brand name, among others, is a chemotherapy drug that is usually used in combination with other drugs to treat different types of cancer, including breast cancer, Hodgkin’s lymphoma, brain cancer, prostate cancer, and testicular cancer [2,3,4,5]. However, its cytotoxicity and nonspecific biodistribution are the major challenges of VBL therapy, as they cause severe side effects in patients [6]. Targeting the drug to tumors can be accomplished by integrating the active ingredient into appropriate nanostructured materials with desirable characteristics of size, surface, and charge [7]. Drug-conjugated superparamagnetic iron oxide (SPION) nanoparticles have been evaluated as a strategy for delivering anticancer agents to tumor sites [8]. The unique characteristics of SPION, such as low toxicity and rapid external magnetic field reaction, have attracted considerable attention from researchers, especially for potential cancer detection and treatment. Moreover, many biocompatible polymers, such as chitosan, polyethylene glycol (PEG), dextran (DEX), and poly-l-lysine (PLL), have been used for the development of SPION nanoparticles as drug and gene delivery systems [9,10,11]. DEX, a polysaccharide, has been widely and successfully utilized for different in vitro and in vivo applications. The coating of SPIONs with DEX offers desirable stability, with no toxicity recorded to date [10]. Furthermore, it provides a robust nanostructured platform for targeted therapeutic delivery [12]. DEX is a natural hydrophilic polymer that can be fully degraded in living biological systems, especially at an acidic pH. This property renders DEX a suitable candidate for controlled drug release purposes [13,14]. FA is overexpressed on the surfaces of different kinds of cancer cells, including liver cancer cells; therefore, it has been employed to target therapeutic agents to cancer cells [15]. These systems allow extensive delivery of local drugs, thus affording an increase in medicine concentrations inside cancer cells, such as the medium and minimum concentrations of drug in the bloodstream and other tissues [16,17]. Gemcitabine and paclitaxel are part of the standard chemotherapy in the treatment of pancreatic cancer. However, no studies have examined the PANC-1 pancreatic cancer cells treated with VBL. Additionally, we prepared a stimuli-responsive controlled VBL drug release system from magnetically sensitive dextran folate composite to enhance the targeted delivery and uptake of vinblastine (VBL) in PANC-1 pancreatic cancer cells. This study developed a stimuli-responsive controlled VBL drug release system based on the FA-DEX-SPION formulation at the nanoscale level, with biocompatible properties for a tumor-specific delivery and incorporating those for pancreatic cancer cells, which are illustrated in (Figure 1).

## 2. Results and Discussion

### 2.1. Characterization of the Synthesized Nanoparticles

#### Morphology and Size of the FA-DEX-VBL-SPION Nanoparticles

**Figure 2:**
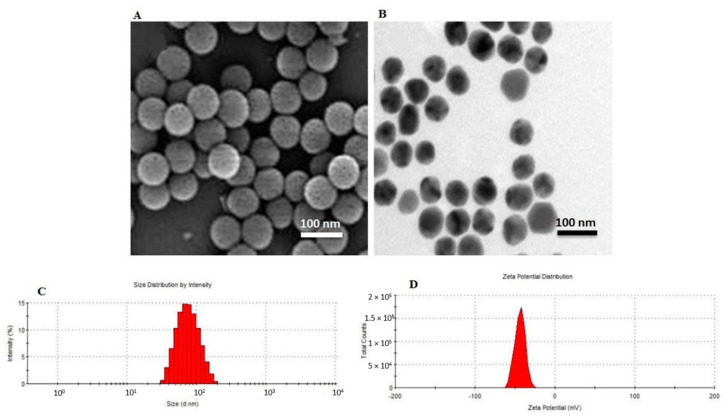
Microscopic analysis and dynamic light scattering (DLS) studies of FA-DEX-VBL-SPION. FESEM image (**A**), HRTEM image (**B**), diameter size (**C**), and surface charge (**D**).

Field emission scanning transmission electron microscopy (FESEM) and High-resolution transmission electron microscopy (HR-TEM) were used to determine the shape and surface morphology of the NPs that were prepared using the optimized formulation. The images from SEM and TEM showed that the NPs were spherical particles with a smooth surface and good dispersity (Figure 2A,B). The particle size and its distribution were measured by DLS (Malvern Zetasizer ZS, Malvern, UK), which showed that the hydrodynamic diameter of NPs was ~ 74 ± 13 nm (Figure 2C). Particle size is among the most relevant parameters in the control of the biocompatible and bioactive properties of the nanoparticles. Particle size is also a critical factor because it has a strong association with stable nanocarrier formulation [18]. Wang et al. prepared similar FA-DEX-SPION nanocarriers with a diameter of 96 nm. In contrast, here, the whole drug-loaded FA-DEX-SPION was almost ~20 nm smaller than in their study [19].

Moreover, the Zeta potential results showed a high negative surface charge of NPs (Figure 2D). These results indicate that the surface-charged nanoparticles were well dispersed in aqueous solution under neutral conditions and, thus, may be applied for cell capture.

### 2.2. FTIR and Magnetization Studies

**Figure 3:**
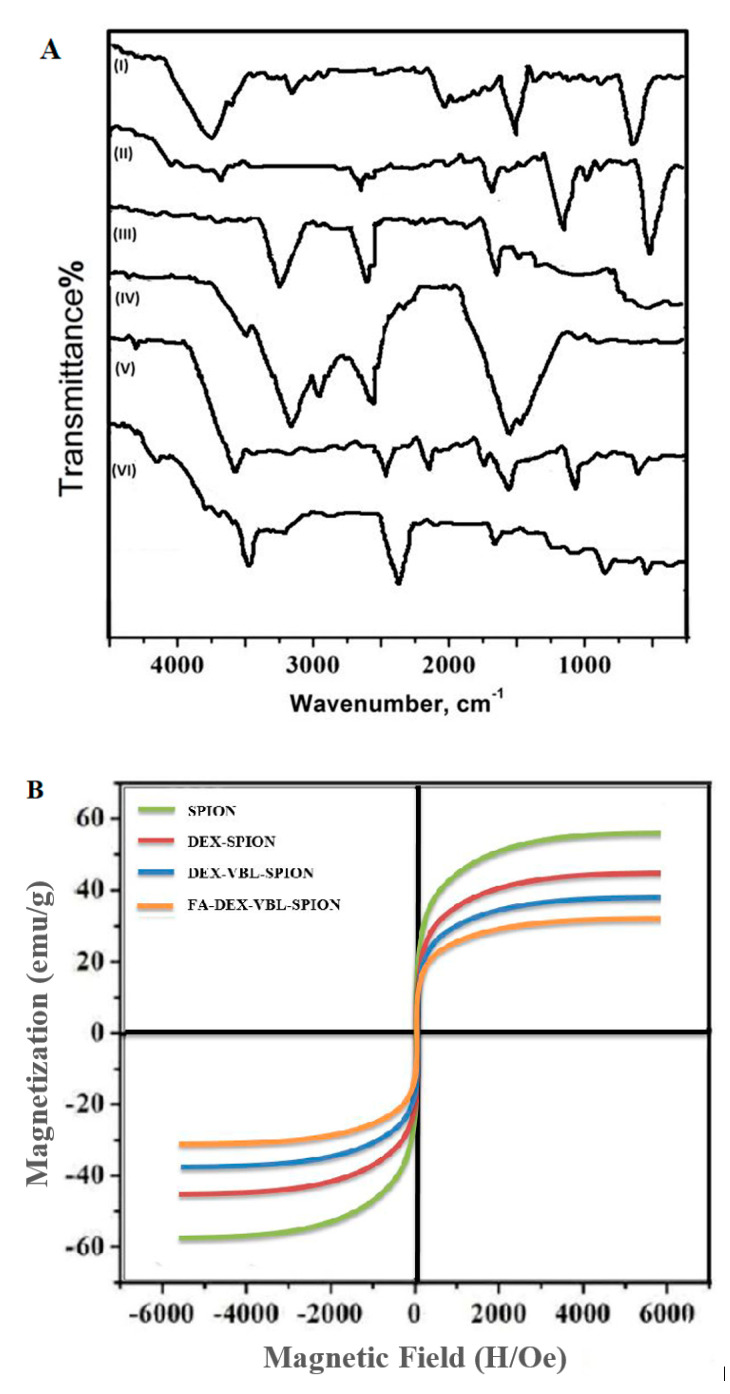
(**A**) FTIR spectra analysis of DEX (I), SPION (II), VBL (III), FA (IV), DEX-SPION (V), and FA-DEX-VBL-SPION (VI). (**B**) Magnetization curve loop of SPION, DEX-SPION, DEX-VBL-SPION, and FA-DEX-VBL-SPION at 300 K.

FTIR was performed to confirm the functional groups on the surface of the synthetic nanoformulation. The spectra of DEX absorption peaks were 3300 and 1600 cm^−1^ because of the OH stretching and HOH-bending modes of the residual water on the particle surface, as shown in Figure 3A (I). The presence of two strong absorption peaks of SPION was observed at around 464.8 and 570.9 cm^−1^ (Figure 3A (II)). The band observed at 592 cm^−1^ was confirmed as the Fe-O stretching vibration of tetrahedral sites of the spinel structure [20]. Figure 3A (IV) shows an infrared spectrum peak of free FA (stretching vibration of the benzene ring skeleton at 1500/cm^−1^). In contrast, in Figure 3A (III), two distinctive absorption peaks at approximately 2930 cm^−1^ (overlapping C-H stretching vibrations of methyl, methylene, and -CH) and 1689 cm^−1^ (stretching vibration peak of the C=O group) were observed, suggesting the loading of VBL within FA-DEX-SPION NPs [21]. The magnetic properties of NPs were examined by vibrating sample magnetometry (VSM). As illustrated in Figure 3B (A), an uncoated SPION, (B) DEX-coated SPION, (C) DEX-VBL-SPION, and (D) FA-DEX-VBL-SPION were prepared, respectively. The saturation magnetization of the uncoated SPION was 55 emu/g, while that of DEX-coated SPION was 43 emu/g, and those of DEX-VBL-SPION and FA-DEX-VBL-SPION were 37 and 30 emu/g, respectively. This decrease in saturation magnetization was attributed to the presence of a large amount of diamagnetic DEX in the SPION nanoformulation [22].

### 2.3. In Vitro Drug Loading and Release Studies

**Figure 4:**
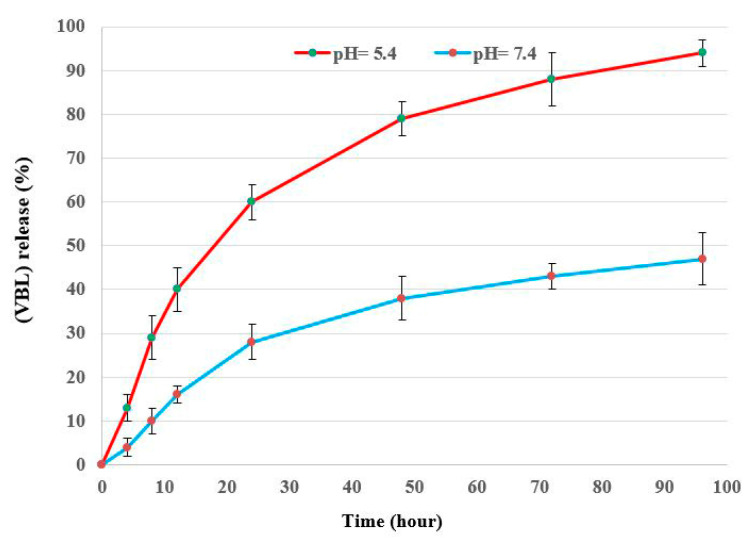
In vitro release profile of the FA-DEX-5-FU-SPION nanoformulation at pH 7.4 and pH 5.4. All experiments were performed at 37 °C. Data are presented as mean values ± SD (*n* = 3).

**Figure 5:**
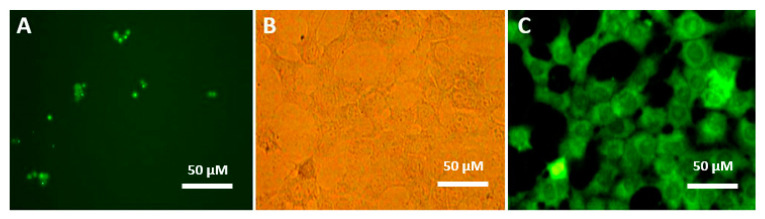
Cell internalization study of FA-DEX-VBL-SPION in the PANC-1 cell line using fluorescence microscopy (400× magnification). Fluorescence microscopy image of VBL-treated cells (**A**). Optic microscopy image of FA-DEX-VBL-SPION-treated cells (**B**). Fluorescence microscopy image of FA-DEX-VBL-SPION-treated cells (**C**).

Figure 4 depict the in vitro drug loading and release profile of VBL from FA-DEX-SPION NPs. The loading profile (Figure 4) shows the rapid adsorption of VBL, followed by the slowing of the adsorption rate 90 min later because the surface of the nanoparticles was covered by VBL [23]. This may be attributed to the type of core-shell structure and the presence of polymer provides several functional groups for a more significant interaction with drug molecules on the drug delivery system’s surface [24]. Based on the in vitro release curves shown in Figure 5, the VBL release time from loaded FA-DEX-SPION occurred over 96 h, and the release rate was faster in citrate buffer (with an acidic pH of 5.4) compared with phosphate buffer (with a normal pH of 7.4) under the same conditions.

### 2.4. Cellular Internalization

The formulated VBL in the FA-DEX-SPION nanocarrier efficiently internalized inside the cancer cells and was visualized clearly by fluorescence microscopy (Figure 5B,C), whereas void VBL aggregated as crystal bodies with different sizes (Figure 5A). PANC-1 cells treated with FA-DEX-VBL-SPION appeared green because of the significant uptake volume due to the enhanced VBL solubility after loading onto the FA-DEX-SPION nanocarrier. In contrast, in cells treated with void VBL, green, star-like, and insoluble particles were visible in the intercellular space because of their insolubility in aqueous milieu [25]. This internalization might be due to folate-receptor-mediated endocytosis [26]. This observation clearly infers that FA-DEX-VBL-SPION is a very effective carrier that can be used as a delivery system for targeted anticancer drugs.

### 2.5. MTT Assay

**Figure 6:**
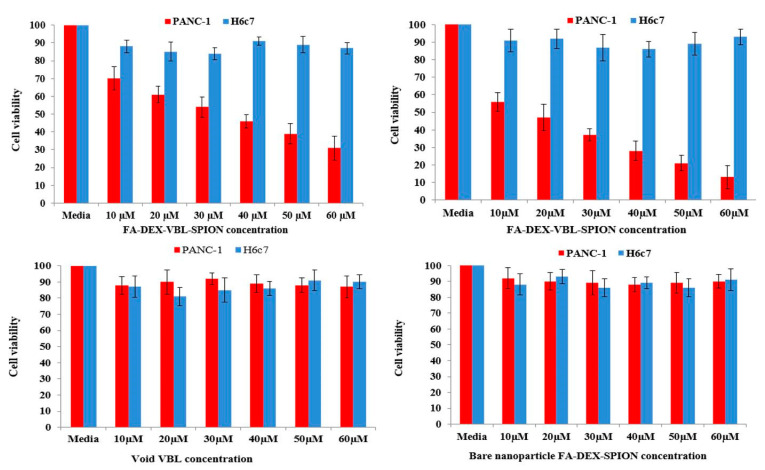
MTT assay of FA-DEX-VBL-SPION, free VBL, and FA-DEX-SPION at 24 h and 48 h on PANC-1 and H6c7 cells.

**Figure 7:**
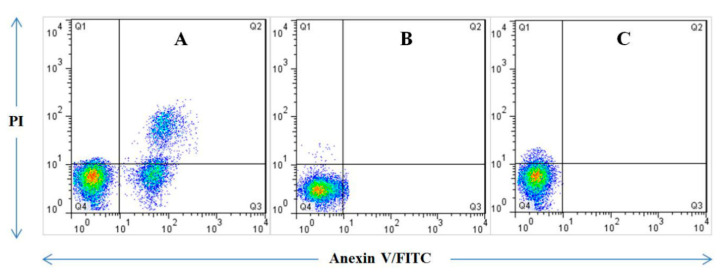
Apoptosis induction by FA-DEX-VBL-SPION nanoformulation. The PANC-1 cell line was treated with (**A**) FA-DEX-VBL-SPION, (**B**) free FA-DEX-SPION, and (**C**) free VBL. The number of PANC-1 cells undergoing apoptosis increased after treatment with the FA-DEX-VBL-SPION nanoformulation. Moreover, treatment of PANC-1 cells with bare FA-DEX-SPION and free VBL separately showed that neither of the treatments yielded a remarkable apoptosis induction.

The cytotoxicity results of the FA-DEX-VBL-SPION were examined by MTT assay against PANC-1 and H6C7 cell lines as shown in Figure 6. The cancer cells were treated with both void VBL and bare FA-DEX-SPION. Even at the highest concentration, 60 μM showed no toxicity for cells, and more than 90% of cells still survived after 48 h of incubation, which indicated they were cytocompatible. Cell viability also decreased significantly when treated with FA-DEX-VBL-SPION and indicated higher inhibition activity of cancer cells as compared with free VBL and bare FA-DEX-SPION nanoparticles alone. IC50 concentration was determined by a dose-response curve fitting of the cell viability date. The PANC-1 cells were stained with annexin V and PI for apoptosis analysis after treatment with free VBL, the FA-DEX-SPION nanocarrier, and FA-DEX-SPION embedded with VBL for 48 h. The cells with no treatment were considered as the control group (Figure 6). Based on the flow cytometric analysis, the blank nanocarrier caused the death of some cells because of hypoxia effects of sedimented nanocarriers on the cells, which suggests that the nanocarrier is safe for use as a drug-delivery system. Furthermore, the apoptotic effects of the FA-DEX-SPION nanocarrier and free VBL were examined; we detected a higher apoptosis rate for the drug-loaded nanocarrier compared with the free drugs. The apoptosis percentage of treated cells is reported in Figure 7. The apoptotic effect was achieved using the same concentration of FA-DEX-SPION. It is well known that apoptosis is an orchestrated cellular process that can occur in physiological and pathological conditions [27]. In cancer, cell proliferation is uncontrolled and apoptosis is suppressed [28]. Moreover, cell proliferation is decreased when cell-cycle arrest occurs. In the condition of DNA damage, cell-cycle arrest is initiated as an attempt to repair the damage; however, if the damage is too extensive for repair, cells die via apoptosis [29].

### 2.6. Gene Expression

**Figure 8:**
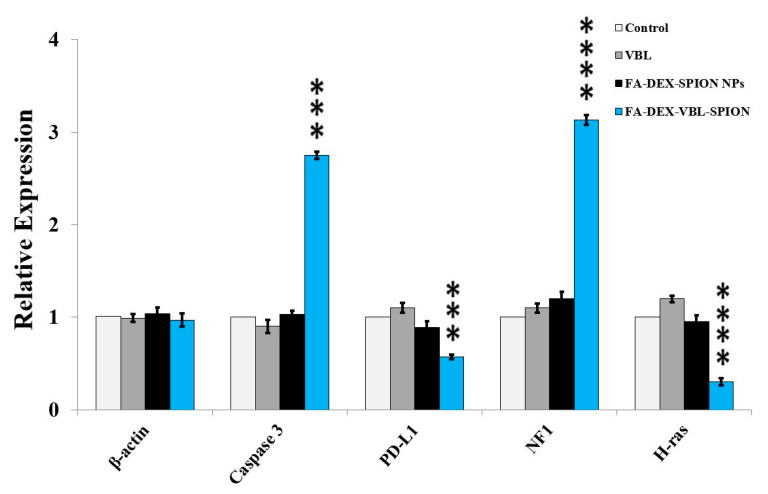
Real-time PCR gene expression analysis of PANC-1 cells treated with void VBL, FA-DEX-VBL-SPION, and FA-DEX-SPION, as assessed using two-way ANOVA and Bonferroni post-test. The values in the graph represent the mean ± SD. *** *p* < 0.001, **** *p* < 0.0001 indicate significant differences between the control (untreated) and other treatments.

Real-time quantitative RT-PCR analysis demonstrated the upregulation of *caspase-3*, *PDL-1*, *NF-1*, and *H-ras* in PANC-1 cells treated with FA-VBL-DEX-SPION, indicating the important role of these selective genes in the present apoptotic process. As mentioned above, the expression levels of the *caspase-3*, *PDL-1*, *NF-1*, and *H-ras* genes were examined using qPCR, and beta-actin was considered as a reference control gene (housekeeping gene). All candidate genes were significantly differentially expressed between malignant and non-malignant samples (Figure 8). The expression levels of beta-actin remained constant among the control and cancerous cells, regardless of the presence or absence of treatment with VBL. However, the expression level of the *caspase-3* gene was clearly increased in cancerous cells after treatment with FA-DEX-VBL-SPION compared with VBL and FA-DEX-SPION (**** *p* < 0.0001). It has been recognized that *NF-1* acts as a tumor suppressor protein [30]. Our results showed that *NF-1* was highly expressed in the PANC-1 cells treated with VBL-loaded DEX-SPION-FA nanoparticles, as shown by qRT-PCR. Successful targeting was delivered to the site of action via an interaction with its receptor on the cell, which indicated its inhibitory effect on cellular tumor growth and proliferation. Conversely, the *PDL-1* and *H-ras* genes play an essential causal role in carcinogenesis, and their low expression in cells represents a reliable indicator of treatment response [31]. We found that low expression levels of the *caspase-3* and *NF-1* genes were visibly reduced in cancerous cells treated with FA-DEX-VBL-SPION compared with void VBL and FA-DEX-SPION, which suggests that the drug was successfully delivered to the site of action of the cancerous cells. Because of the novelty of this work, no data have been reported regarding the VBL up- and downregulating effects on the *caspase-3*, *PDL-1*, *NF-1*, and *H-ras* genes in different types of cancer.

## 3. Materials and Methods

### 3.1. Materials

VBL sulfate, DEX (15 kDa), ferric chloride hexahydrate dimethyl sulfoxide (DMSO), FeCl_3_·6H_2_O, ferrous chloride tetrahydrate (FeCl_2_·4H_2_O), the MTT agent, NH_4_OH (25% of ammonia), and FA were purchased from Merck (Darmstadt, Germany). Annexin V-FITC, propidium iodide (PI), trypan blue, 96-well cell culture plates, and cell culture flasks were purchased from Sigma-Aldrich (St. Louis, MO, USA). The PANC-1 and H6c7 cell lines were obtained from the American Type Culture Collection (ATCC, Manassas, VA, USA).

### 3.2. Fabrication of DEX-SPION

SPION was fabricated by the coprecipitation method, as described previously, with some modification [32]. Briefly, N_2_ gas was poured into 55 mL of distilled water. Next, 2 mmol of FeCl_2_·4H_2_O, 4 mmol of FeCl_3_·6H_2_O, and 10 mL of 0.5% DEX were added to the solution, followed by the addition of NH_4_OH in a dropwise manner using a syringe. Subsequently, the mixtures were stirred at 65 °C for 30 min under a nitrogen atmosphere and centrifuged. The resulting black precipitates were collected.

### 3.3. Preparation of VBL-Loaded FA-DEX-SPION

Briefly, 100 mg of prepared DEX-SPION was added to 20 mg of VBL (previously dissolved in 20 mL of DMSO) and stirred for 24 h. Subsequently, FA functionalization of this nanoformulation was performed by adding 5 mg/mL of this molecule to the obtained mixture (FA-DEX-VBL-SPION) [33]. The surface functionalization of DEX-VBL-SPION was performed because of electrostatic reactions. The FA-DEX-VBL-SPION was detached by centrifugation at 15,000 rpm and then rewashed three times with distilled water. A vacuum oven was used to dry the VBL-entrapped nanoparticles at 40 °C for 8 h. The unloaded VBL was calculated by measuring its concentration in the supernatant using a UV-Vis spectrophotometer at a wavelength of 425 nm. The efficacy of drug encapsulation was calculated using the following Equation (1) [34].

(1) $$\mathrm{Encapsulation}\;\mathrm{Efficiency}\;\left(\%\right)=\frac{\mathrm{Tatal}\;\mathrm{quantity}\;\mathrm{of}\;\mathrm{drug}-\mathrm{Free}\;\mathrm{quantity}\;\mathrm{of}\;\mathrm{drug}}{\mathrm{Total}\;\mathrm{amount}\;\mathrm{of}\;\mathrm{drug}}\times 100$$

### 3.4. Characterization of NPs

The size and morphological features of the obtained FA-DEX-VBL-SPION nanocomposite were estimated using TEM (Zeiss EM900, Carl Zeiss AG, Jena, Germany) and SEM (Hitachi S-3000 SEM, Tokyo, Japan) at a voltage of 30.0 kV and 30 mA. Zeta potential and dynamic light scattering (DLS) (Nano S, Malvern, UK) were performed after resuspension in ultrapure water and dilution to an appropriate concentration. FTIR spectra were recorded on a Thermo Nicolet 6700 instrument (AEM, Madison WI, USA) within the range of 400–4000 cm^−1^. Moreover, VSM (Lakeshore 7404, LakeShore, MI, USA) was performed to evaluate the magnetic properties of the particles.

### 3.5. Measurement of Drug Release

VBL release from FA-DEX-SPION was performed in phosphate-buffered saline (PBS) (0.01 M, pH = 7.4) and citrate buffer (0.01 M, pH = 5.4) at 37 °C. One milliliter of the drug-loaded micellar solution was poured into two separate dialysis bags (Spectra/por; MW cutoff, 3500 gmol^−1^). These bags were placed in phosphate (100 mL, 0.01 M, pH 7.4) and citrate (100 mL, 0.01 M, pH 5.4) buffers separately. Tween 80, as an emulsifying agent, was added to each of these buffer solutions to inhibit the possible sedimentation of the released drug. The temperature was fixed at 37 °C, and buffers were stirred gently on a shaker (GFL, Burgwedel, Germany). Sampling was performed at 0, 4, 8, 12, 24, 48, 72, and 96 h. At each time point, 500 μL of the specimen was collected and subjected to freeze-drying. The residue was dissolved in 2 mL of methanol. The quantity of released VBL was determined by nano-drop. Drug release was measured using the following Equation (2):

(2) $$R=\;\frac{V\sum_{i}^{n-i}Ci+Vo\;Cn}{m{\;}_{drug}}$$

where *R* is the drug release accumulation (%), *V* is the sampling volume, *V*_0_ is the first volume of the drug, *C_i_* and Cn are the VBL concentrations, *i* and *n* are the sampling times, and *m_drug_* is the mass of the VBL loaded onto FA-DEX-SPION. The precipitated material was rinsed and resuspended in DDW.

### 3.6. Cell Culture Conditions

Pancreatic cancer (PANC-1 and H6c7) cell lines were incubated in culture medium (DMEM) supplemented with 10% fetal bovine serum (FBS) and penicillin/streptomycin (Gibco, Life Technologies, Paisley, UK) and grown in a humidified incubator of 5% CO_2_ at 37 °C (Plymouth, MN, USA).

### 3.7. Cell Internalization Assay

FA-DEX-VBL-SPION was functionalized with fluorescein 5(6)-isothiocyanate (FITC) to evaluate its cell internalization efficiency using a fluorescence microscope (Nikon Eclipse TE2000-U). The Fluorescein Isothiocyanate (FITC)-dextran was synthesized by coupling fluoresceinyl isothiocyanate (5-isomer) to dextran polymer. FITC was conjugated randomly to hydroxyl groups of dextran at a frequency of 0.003 to 0.02 moles of FITC per mole of glucose. The cells were treated with 5 μg of FITC-FA-DEX-VBL-SPION for 3 h. Subsequently, the nanocomposite-containing medium was discarded, and the cells were washed with PBS. Photomicrographs were acquired using a fluorescence microscope (Nikon Eclipse TE2000-U).

### 3.8. MTT Assay

For the MTT assay, 200 μL of medium containing 1 × 10^4^ cells was poured into each well of a 96-well plate. The cells were allowed to adhere and grow for 24 h. The medium of each well was removed and replaced with fresh medium containing varying concentrations (10 to 60 μM) of either VBL, FA-DEX-VBL-SPION, or FA-DEX-SPION, followed by incubation for 24 and 48 h. A group of cells without treatment was used as the control. A suitable concentration of MTT solution (10 µL of a 5 mg/mL solution in each 100 µL of medium) was added to each well. The plates were incubated at 37 °C in a humidified incubator containing 95% air and 5% CO_2_ for 4 h. The remaining MTT solution was removed and 100 µL of DMSO was added to each well, to dissolve the formazan crystals. The plates were shaken for 5 min to ensure that the formazan crystals dissolved adequately. The absorbance in each well was recorded at 540 nm using a multiscan plate reader (VERSAmax microplate reader, Molecular Device, CA, USA). The results are presented as the mean ± SD.

Relative cell toxicity = [(A_sample_ − A_control_)/A_control_] × 100 (3)

### 3.9. Apoptosis Assay by Flow Cytometry

A flow cytometry assay was applied to estimate the average of apoptosis and necrosis in cells exposed to different treatments with the nanodrug composite, void VBL, and FA-DEX-VBL-SPION for 48 h and stained with Annexin V-FITC and propidium iodide (PI). Subsequently, the cells were harvested using trypsin, counted, and then poured into 6-well plates for ~10^4^ cells/well. Apoptosis was estimated using the Annexin V-FITC Apoptosis Detection Kit (Biovision, Inc., Mountain View, CA, USA) according to the manufacturer’s protocols.

### 3.10. RT-PCR

Forty-eight hours after PANC-1 cell treatment, the total RNA was extracted from cell lysates using TRIzol (Invitrogen Life Technologies, Paisley, UK). The concentrations and quantity of RNA were determined by measuring OD (260/280 nm wavelength). Total RNA was classically used in the cDNA synthesis kit (Fermentas, Germany). According to the instructions of the manufacturer of the kit, five pairs of oligonucleotide primers for targets and endogenous genes were used and referenced [35,36,37,38,39], as illustrated in Table 1.

### 3.11. Real-Time PCR

To estimate the expression levels of the *caspase-3*, *PD-L1*, *NF-1*, and *H-ras* genes, real-time PCR was carried out on an ABI prism instrument (Applied Biosystems, Forster City, CA, USA). Beta-actin was considered as the reference control gene. Amplification reactions contained 5 μL of cDNA, 10 μL of the SYBR Green-I dye (Applied Biosystems), and 0.5 μL of each specific primer. PCR was carried out as follows: initial denaturation at 95 °C for 10 min; followed by 50 cycles of 95 °C for 15 s and 60 °C for 1 min. The real-time PCR success was examined via a melting curve analysis.

## 4. Conclusions

Dextran-coated superparamagnetic nanoparticles (DEX-SPION) conjugated with FA and carrying the anticancer drug VBL were successfully fabricated via a co-precipitation approach. The nano vehicle improved the drug loading with nanoscale particle size distribution in PANC-1 cancer cells. The developed FA-DEX-VBL-SPION nanocarrier exhibited a sustainable release profile, leading to dose- and time-dependent targeted pancreatic cell cytotoxicity. FA-DEX-VBL-SPION had a stronger inhibitory activity against tumor growth in PANC-1 pancreatic cancer cells than did VBL alone and FA-DEX-SPION. Moreover, FA-DEX-VBL-SPION showed high biocompatibility, loading efficiency, controllability, and penetrability, which render it a useful and exciting tool for a wide range of potential applications in biomedicine. We report that FA-DEX-VBL-SPION could induce cytotoxic effects on the *caspase-3*, *PDL-1*, *NF-1*, and *H-ras* gene expression levels, and lead to a reduction in cancerous cells, thereby effectively controlling cancer progression without toxicity to healthy cells compared with void VBL and FA-DEX-SPION. Therefore, this nanocarrier might be applied as a safe and active antitumor factor that could be used in clinical applications.

## Figures and Tables

**Table 1:** Primers used for *β-actin*, *caspase-3*, *PD-L1*, *NF-1*, and *H-ras* gene amplification in the present study.

Primer Name	Primer Sequence Oligo Sequence F (5’→3’)	Primer Sequence Oligo Sequence R (5’→3’)	Ref.
*β-actin*	CTGGCACCCAGCACAATG	GCCGATCCACACGGAGTACT	[35]
*Caspase* *-3*	CATACTCCACAGCACCTGGTTA	CGCAAAGTGACTGGATGAACC	[36]
*PD-L1*	TGTGAAAGTCAATGCCCCAT	TGTCAGTTCATGTTCAGAGGT	[37]
*NF-1*	CGCAGCAGCACCCACATTTAC	ACTGTGGCGGGGACTCCTCA	[38]
*H-ras*	TTCTACACGTTGGTGCGTGA	CACAAGGGAGGCTGCTGAC	[39]
